# Monitoring mepolizumab treatment in chronic rhinosinusitis with nasal polyps (CRSwNP): Discontinue, change, continue therapy? 

**DOI:** 10.5414/ALX02460E

**Published:** 2024-03-21

**Authors:** Ludger Klimek, Ulrike Förster-Ruhrmann, Heidi Olze, Achim G. Beule, Adam M. Chaker, Jan Hagemann, Tilman Huppertz, Thomas K. Hoffmann, Stefan Dazert, Thomas Deitmer, Sebastian Strieth, Holger Wrede, Wolfgang W. Schlenter, Hans-Jürgen Welkoborsky, Barbara Wollenberg, Sven Becker, Fredericke Bärhold, Felix Klimek, Ingrid Casper, Jaron Zuberbier, Claudia Rudack, Mandy Cuevas, Constantin A. Hintschich, Orlando Guntinas-Lichius, Timo Stöver, Christoph Bergmann, Pascal Werminghaus, Oliver Pfaar, Jan Gosepath, Moritz Gröger, Caroline Beutner, Martin Laudien, Rainer K. Weber, Tanja Hildebrand, Anna S. Hoffmann, Claus Bachert

**Affiliations:** 1 Center for Rhinology and Allergology, Wiesbaden,; 2 HNO-University Clinic Charité, Berlin,; 3 Clinic for Otorhinolaryngology, Münster University Hospital,; 4 Clinic for Otorhinolaryngology, Head and Neck Surgery at Greifswald University Medical Center,; 5 Department of Otolaryngology - Head and Neck Surgery, Klinikum rechts der Isar, Technical University of Munich, Munich,; 6 Center of Allergy and Environment (ZAUM) of the Technical University of Munich,; 7 Clinic and Polyclinic for Otolaryngology, University Medical Center Mainz, Mainz,; 8 Department of Otorhinolaryngology, Head and Neck Surgery, University of Ulm, Ulm,; 9 Clinic for Otorhinolaryngology, Head and Neck Surgery, Ruhr University Bochum, St. Elisabeth Hospital,; 10 German Society for Otorhinolaryngology, Head and Neck Surgery, Bonn,; 11 Clinic and Polyclinic for Otorhinolaryngology, University Hospital Bonn,; 12 Ear, nose and throat specialist, Herford,; 13 Medical Association of German Allergists, Wiesbaden,; 14 Clinic for Ear, Nose and Throat Medicine, Head and Neck Surgery, Nordstadt Clinic of the KRH, Hannover,; 15 HNO-University Clinic Tübingen,; 16 Clinic and Polyclinic for Otolaryngology, University Hospital Carl Gustav Carus, TU Dresden, Dresden,; 17 Clinic and Polyclinic for Ear, Nose and Throat Medicine, University Hospital Regensburg, Regensburg,; 18 Clinic for Otorhinolaryngology, Jena University Hospital,; 19 Otorhinolaryngology University Clinic Frankfurt am Main,; 20 HNO RKM740 Interdisciplinary Specialist Clinic, Düsseldorf, Germany,; 21 Praxis für Hals-, Nasen-, Ohrenheilkunde und Allergologie, Düsseldorf,; 22 Clinic for Ear, Nose and Throat Medicine, University Hospital Giessen and Marburg GmbH, Marburg site, Philipps University Marburg, Marburg,; 23 Clinic for Otorhinolaryngology, HSK Wiesbaden,; 24 Clinic and Polyclinic for Otorhinolaryngology, University Hospital LMU Munich,; 25 Department of Dermatology, Venereology and Allergology, University Medical Center Göttingen, Germany Klinik für Dermatologie, Venerologie und Allergologie, Universitätsmedizin Göttingen,; 26 Department of Otorhinolaryngology, Head and Neck Surgery, Kiel University, University Medical Centre Schleswig-Holstein, Kiel,; 27 Clinic for Otorhinolaryngology, Karlsruhe Municipal Hospital,; 28 Department of Ear, Nose and Throat Medicine, Freiburg University Medical Center,; 29 Clinic for Ear, Nose and Throat Medicine, University Medical Center Hamburg-Eppendorf,; 30 Upper Airways Research Laboratory and Department of Oto-Rhino-Laryngology, Ghent University and Ghent, University Hospital, Ghent, Belgium

**Keywords:** chronic rhinosinusitis, CRSwNP, biologicals, eosinophilic inflammation, mepolizumab

## Abstract

Background: Chronic rhinosinusitis with nasal polyps (CRSwNP) is a multifactorial inflammatory disease of the mucous membranes of the nose and sinuses. Eosinophilic inflammation is described as a common endotype. The anti-IL-5 antibody mepolizumab was approved in November 2021 as an add-on therapy to intranasal glucocorticosteroids for the treatment of adults with severe chronic rhinosinusitis with nasal polyps when systemic glucocorticosteroids or surgery do not provide adequate disease control. While national and international recommendations exist for the use of mepolizumab in CRSwNP, it has not yet been adequately specified how this therapy should be monitored, what follow-up documentation is necessary, and when it should be discontinued if necessary. Materials and methods: A literature search was performed to analyze previous data on the treatment of CRSwNP with mepolizumab and to determine the available evidence by searching Medline, Pubmed, the national and international trial and guideline registries, and the Cochrane Library. Human studies published in the period up to and including 10/2022 were considered. Results: Based on the international literature and previous experience by an expert panel, recommendations for follow-up, adherence to therapy intervals, and possible therapy breaks as well as discontinuation of therapy when using mepolizumab for the indication CRSwNP in the German healthcare system are given on the basis of a documentation sheet. Conclusion: Understanding the immunological basis of CRSwNP opens up new non-surgical therapeutic approaches with biologics for patients with severe, uncontrolled courses. Here, we provide recommendations for follow-up, adherence to therapy intervals, possible therapy pauses, or discontinuation of therapy when mepolizumab is used as add-on therapy with intranasal glucocorticosteroids to treat adult patients with severe CRSwNP that cannot be adequately controlled with systemic glucocorticosteroids and/or surgical intervention.

## Introduction 

### Chronic rhinosinusitis with nasal polyps 

Chronic rhinosinusitis with nasal polyps (CRSwNP) is characterized by nasal obstruction, a feeling of pressure in the paranasal sinuses, loss of smell, and anterior and/or posterior rhinorrhea. 

All the drug and surgical treatments available to date do not provide sufficient disease control and relapse prevention in some cases [1]. Oral glucocorticosteroid (GCS) therapy is often used to control exacerbations, although in the past steroid-related side effects had to be tolerated.. There is therefore an unmet need for new treatments to better control the disease. Advances in the understanding of the immunological processes involved in type 2 inﬂammation, which underlies ~ 80% of cases of CRSwNP in Europe [2, 3] and the USA [4, 5], have led to new possibilities for disease control. Monoclonal antibodies targeting eosinophilic or type 2 inflammation are also available in Europe for the treatment of CRSwNP with mepolizumab, omalizumab, and dupilumab. 

International recommendations [1, 6, 7, 8], consensus recommendations for the German healthcare system [9, 10] have been developed for the use of dupilumab [11, 12], omalizumab [13, 14], and mepolizumab [15] including special considerations during the COVID-19 pandemic [16]. 

In this position paper, we provide recommendations for monitoring the course and efficacy of mepolizumab therapy, its duration, and possible discontinuation of therapy. It is based on both the pivotal phase 3 trials that led to the approval of mepolizumab and the advancing knowledge about the use of mepolizumab in routine care. The safety proﬁle, treatment in the context of different paradigms are considered and pharmacoeconomic aspects taking into account the high costs of biologics in a cost-effectiveness analysis. 

### Prevalence of nasal polyps, pathophysiology, current treatment 

Chronic rhinosinusitis (CRS) is the second most common chronic disease in Europe and the USA [4]. CRSwNP, the most severe subtype of CRS, accounts for the majority of the healthcare costs of CRS with a prevalence of ~ 4% in the adult population [4] and is associated with a significantly impaired health-related quality of life [17, 18]. CRSwNP often recurs despite adequate medical and surgical treatment [19]. The main symptoms include obstructed nasal breathing, loss of sense of smell, a feeling of pressure around the face and anterior and posterior rhinorrhea [7, 20, 21, 22]. 

Drug treatment of CRSwNP targets the underlying inflammation with symptoms as described above. Standard treatments include topical intranasal corticosteroids, short-term treatment with systemic corticosteroids (SCS) and endoscopic sinus surgery [7, 21, 23]. Treatment also includes nasal rinsing with brine solutions and antibiotics to treat any acute bacterial exacerbations. Sinus surgery is an option for patients whose symptoms persist despite appropriate medication [7]. However, in addition to sinus surgery, drug treatment is always continued, primarily in the form of intranasal GCS and nasal rinsing. 

Patients with severe CRSwNP have a recurrence rate of 40% within 3 years, even when multimodal treatment methods are used [24], and up to 80% within 12 years [24, 25, 26]. Therefore, additional treatment options are needed. 

Patients with severe CRSwNP and comorbid asthma, aspirin-exacerbated airway disease (AERD), or eosinophilic inflammation are most affected by the disease. Importantly, these patients require sinus surgery more frequently, have a high corticosteroid consumption and are more likely to relapse in the long term than patients without these disease characteristics [24, 26, 27, 28, 29]. Patients with asthma and AERD account for 23 – 45% [30, 31, 32] and 10 – 16% [29, 31] of patients with severe CRSwNP, respectively. Biologic therapy targets type 2 inflammation, which is found in ~ 80% of patients with CRSwNP in Europe. Indications of type 2 inflammation are severe refractory CRSwNP, comorbid asthma, and an eosinophil count of more than 300 cells/µL in the blood [33]. In addition, previous mepolizumab studies in severe eosinophilic asthma indicate that patients with a blood eosinophil count of ≥ 150 cells/µL at baseline were more likely to benefit from mepolizumab therapy [34, 35], although blood eosinophil count has not yet been established as a clear biomarker for the efficacy of mepolizumab in CRSwNP [36]. 

### Mechanism of action of mepolizumab 

Advances in understanding the pathogenesis and immunological basis of CRSwNP [3] have enabled the development of monoclonal antibodies as drugs (biologics) for this disease. In CRSwNP, chronic inflammation is primarily determined by type 2 proinflammatory cytokines such as IL-5, IL-4, and IL-13 as well as a high number of eosinophils in the surrounding tissue [7, 37]. CRSwNP is characterized by a disturbed barrier function of the epithelium and often by a type 2 inflammatory pattern, which is also observed in a similar form in bronchial asthma [38]. The activation of T lymphocytes and epithelial cells leads to the release of epithelial cytokines such as IL-25, IL-33, and thymic stromal lymphopoietin [39]. These cytokines activate type 2 innate lymphoid cells (ILCs), adaptive T helper cells, dendritic cells, and mast cells in the tissue and promote type 2 inflammation. The subsequent type 2 immune responses are characterized by the production of IL-4, IL-5, and IL-13 by ILC2, Tc2 (CD8+ T cells expressing the prostaglandin DP2 receptor CRTH2), and Th2 cells. IL-13 contributes significantly and directly to the hyperplasia of goblet cells in the respiratory epithelium and clinically often to dyscrinia and hypersecretion. IL-4 and IL-13 mediate a class switch to IgE production by B cells so that, with increased activity of these cytokines in the diseased tissue in asthma and CRSwNP, IgE antibodies are not an expression of allergy but also of generic type 2 activation. IL-5 recruits eosinophils into the tissue. The increase in T cells, B cells, and plasma cells in the tissue with high IgE concentrations in the mucosa characterizes this inflammatory reaction, which is further intensified by the activation of mast cells and eosinophils. Elevated levels of IL-4 and IL-13, observed proximally in the inflammatory cascade, and IL-5 and eosinophils, observed distally, are considered to be hallmarks of the type 2 inﬂammatory response in polyp tissue [40, 41, 42, 43]. Therefore, these key cytokines have become targets for various monoclonal antibodies as therapeutic agents (biologics). 

Mepolizumab was developed for the treatment of type 2-dependent eosinophilic diseases [44]. Proof of efficacy of mepolizumab in eosinophilic airway diseases was provided for severe eosinophilic bronchial asthma [45] with subsequent global approval in 2015 [46, 47]. 

Mepolizumab is a humanized anti-IL-5 monoclonal antibody that prevents the binding of IL-5 to its receptor on eosinophil granulocytes, mast cells, and other target cells and selectively inhibits eosinophilic inflammation [48]. 

### Mepolizumab in chronic rhinosinusitis with nasal polyps 

Mepolizumab (100 mg administered subcutaneously) is approved in several countries worldwide for the treatment of severe eosinophilic asthma and CRSwNP and in a dose of 300 mg for patients with eosinophilic granulomatosis with polyangiitis (EGPA) and hypereosinophilic syndrome [49, 50, 51]. 

The pivotal study for mepolizumab in CRSwNP was the SYNAPSE study – a randomized, double-blind, placebo-controlled, parallel-group phase 3 study [52]. Detailed information on this study and the approval criteria has been published [53]. 

The phase 3 SYNAPSE study showed that mepolizumab reduced the size of nasal polyps and improved symptoms of nasal obstruction, reduced the actual number of sinus surgeries and the use of SCS, improved sinonasal symptoms, and had an acceptable safety profile [53]. In addition, the initial results of SYNAPSE indicated that mepolizumab improved nasal obstruction in patients with high blood eosinophil counts [53], which was explained by the IL-5-binding and eosinophil-blocking mechanism of action of mepolizumab [48]. Similar observations were made in patients with asthma and chronic obstructive pulmonary disease [34, 54]. 

An exploratory analysis evaluated the efficacy of mepolizumab compared to placebo in adults with severe, bilateral CRSwNP requiring revision surgery depending on the presence of comorbid asthma, comorbid AERD, and blood eosinophil count [55]. However, the extent to which the blood eosinophil count can serve as a possible biomarker for treatment success can currently not be deduced from the available data on CRSwNP [36]. 

### Adverse effects and safety of mepolizumab 

The safety of mepolizumab is well established based on the collective collection of safety data in several phase 3 clinical trials not only for CRSwNP, but also for asthma and hypereosinophilia syndrome, EGPA, and in post-marketing surveillance [53, 56, 57, 58, 59]. 

In general, mepolizumab was well tolerated in the studies presented, and no serious adverse effects occurred [60]. 

Vital signs, physical examinations, laboratory tests, and electrocardiograms were monitored in all studies and provided no evidence of adverse effects. With regard to CRSwNP, the tolerability profile from previous studies was confirmed in the SYNAPSE study. Serious adverse events occurred in 12 (6%) patients on mepolizumab and 13 (6%) on placebo. The overall proportion of patients in whom adverse events were documented also did not differ between the mepolizumab group (169 (82%)) and the placebo group (168 (84%)). The most common adverse events in the study in both patient groups were nasopharyngitis, headache, epistaxis, and back pain [53, 61]. Systemic allergic reactions (type 1 hypersensitivity reactions) were reported in 2 patients (< 1%) in the group receiving mepolizumab 100 mg and in none of the patients in the placebo group [61]. 

### Dosage of mepolizumab in chronic rhinosinusitis with nasal polyps 

Mepolizumab is applied subcutaneously and administered to patients every 4 weeks in a dose of 100 mg by the treating physician – but also by the patients themselves. 

After repeated subcutaneous administration, there was an ~ 2-fold accumulation at steady state with a bioavailability of 80% [62]. The effects of mepolizumab on eosinophils in the blood served as a pharmacodynamic parameter. In patients with CRSwNP, the number of eosinophils in the blood decreased from a geometric mean at baseline of 390 (n = 206) to 60 cells/μL (n = 126) by week 52 after a 100-mg dose of mepolizumab administered subcutaneously every 4 weeks for 52 weeks, which corresponds to a reduction in the geometric mean of 83% compared to placebo. This level of reduction was observed within 4 weeks of treatment initiation and was maintained throughout the 52-week treatment period, demonstrating the onset of action and efficacy of mepolizumab on eosinophils [53, 62]. The current dosing recommendations state that if a dose is missed, it can be made up immediately. If the omission is only noticed at the time of the next dose, only the next dose is injected as planned and the missed dose must be omitted [63]. 

There is currently insufficient data on the question of patient adherence to treatment for routine applications, and this will have to be investigated over time. There is also a lack of data on a possible extension of the injection interval. 

### Evaluation of the initial clinical response to mepolizumab in chronic rhinosinusitis with nasal polyps 

Once therapy with mepolizumab has been initiated for the treatment of uncontrolled, severe CRSwNP, it is important to monitor the patient’s response to the drug. Depending on the clinical endpoint used, non-responders can be expected in ~ 25% of cases in CRSwNP [6, 64]. 

An international panel of experts has issued recommendations for the assessment of the initial response to therapy and the subsequent follow-up [6], on which the information provided here for the German healthcare system is based. We follow the principles of medical treatment in the German social insurance system (economical, appropriate, expedient) to achieve targeted and effective therapy. In particular, this should also avoid inappropriate treatment and the associated unnecessary costs. 

**Figure 1:**
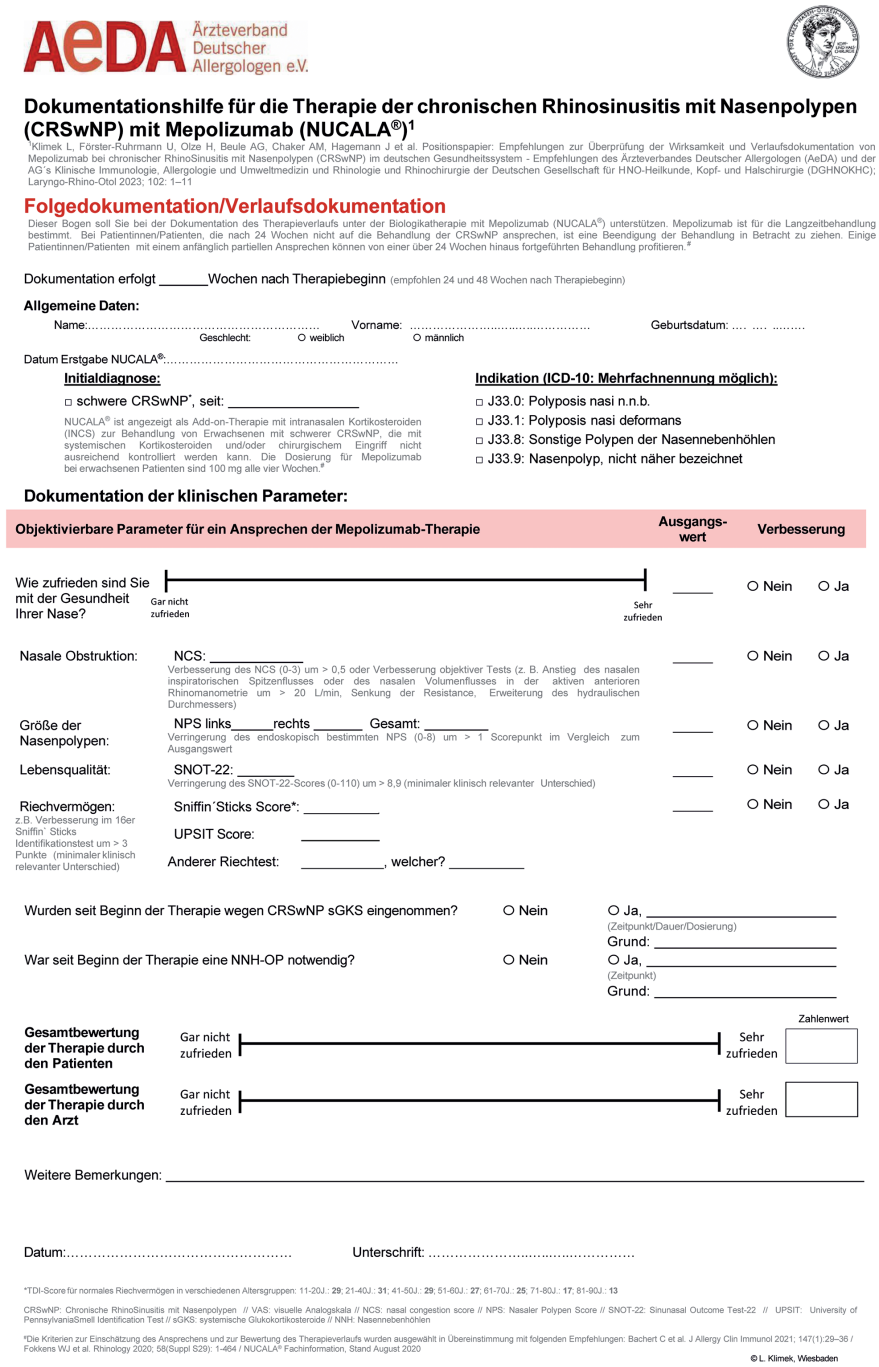
Documentation form for the German healthcare system.

To this end, we provide the following recommendations for the different treatment phases of mepolizumab therapy in CRSwNP, which can be documented in the documentation sheet for monitoring the course of therapy (Figure 1). 

### A response to treatment with mepolizumab in chronic rhinosinusitis with nasal polyps is expected within 4 – 6 months 

As already explained, steady-state concentrations for mepolizumab are reached after approximately 4 weeks of treatment at the recommended doses and injection intervals [53, 62]. We recommend evaluating the success of therapy after 4 – 6 months. 

In the event of a non-response with regard to individual or all parameters, treatment should be continued and re-evaluated after a further 6 months. Both the updated German AWMF guideline [10] and the expert information recommend an assessment of treatment response after 24 weeks [65], and this recommendation for treatment evaluation 6 months after the start of treatment was also made internationally by a European expert group [6]. In contrast to the international position papers, the German guideline does not specify a fixed point in time for the review of efficacy and thus enables more flexible handling and strengthens the treating physician’s therapeutic freedom [10]. 

The chance that a response to treatment (reduction in disease burden) will still occur after 24 weeks / 6 months in the absence of an effect with adequately administered mepolizumab treatment is low [6]. However, the data situation based on prospectively collected data is limited due to the recent approval. 

Within the first 6 months, no other concomitant medication (e.g., oral GCS) or surgery should be combined with mepolizumab other than topical GCS in order to be able to distinguish a response from a non-response, with the exception of emergency treatments and exacerbations. If additive immunosuppressive or immune-modifying therapy was necessary during this period, a delayed efficacy assessment at a later date may be appropriate. 

The treatment effect is defined as the changes in nasal polyp score, olfaction, and symptoms shown in Table 1 using objectifiable symptom- and endoscopy-based criteria. For individual patients, the decision to continue or discontinue treatment is made on the basis of these criteria (Table 1). 

If there is insufficient response to treatment, the treatment strategy should be adapted accordingly, taking into account the patient’s wishes (surgical intervention or switch to another biologic or other therapy, such as short-term administration of systemic GCS) and, if necessary, the patient should be referred to a rhinology center. Surgical intervention should be considered, particularly in the case of a laterally asymmetrical response, if only to rule out secondary pathologies. Locally limited residual polyp findings are possible after administration of biologics and, if functionally relevant, can also be addressed surgically. There is currently no experience available that would sufficiently substantiate a recommendation to switch to a specific biologic after the unsuccessful use of a primary biologic. Therefore, the same criteria should be used for selection as for primary initial therapy [12, 14, 15]. However, it seems logical to switch between the treatment principles of anti-IL-5, anti-IgE and anti-IL-4R. Prospectively collected data would be desirable here. 

Up to now, there is also insufficient data on whether continued biologic therapy in addition to sinus surgery improves the recurrence rate of uncontrolled CRSwNP after only a partial initial response to biologics. In future studies, it should be systematically and prospectively investigated whether continued treatment with mepolizumab can also prevent polyp recurrence after surgery in these patients in the long term and thus allow long-term disease control to be maintained after initial surgical treatment. Further monitoring of the various parameters should also be carried out during the course of treatment in order to detect a decline in the effectiveness of the therapy. 

After 12 months of treatment, a controlled state of CRSwNP with low symptom burden should be achieved so that treatment can be maintained in the following years. Criteria for an adequate response after 12 months of treatment with mepolizumab are presented in Table 2. 

If there is no sufficient response to the biologic therapy after 12 months of treatment (see definition in Table 2), we recommend stopping mepolizumab therapy. After a critical re-evaluation of the clinical indication criteria and the nature of the underlying inflammation (endotype), a switch to a different biologic may be considered or additively a re-operation or short-term therapy with systemic GCS [66]. 

However, as soon as at least one of these criteria is met, the patient is satisfied with the treatment, and no relevant side effects have occurred, treatment with the biologic can be continued. 

### Special instructions for the measurement of olfaction in the German healthcare system 

In the approval studies for mepolizumab, the UPSIT test and the brief smell identification test (B-SIT) were used to objectify the ability to smell [67]. However, these tests are rarely used in routine care in Germany. In contrast, the Sniffin’ Sticks olfactory test [68, 69], a subjective, orthonasal olfactory test procedure, is widely used. Felt-tip pens filled with odorants are used as a test instrument [68]. For a precise assessment of the overall olfactory capacity, the sum of the olfactory threshold, odor discrimination, and odor identification tests is formed resulting in the so-called SDI (Schwellenwert, Diskriminationswert, Identifikationswert – threshold value, discrimination value, identification value). The examination of the SDI is complex and usually only carried out in specialized centers. Nevertheless, the ability to smell should be measured and not just asked for [70, 71]. The identification test with 12 or 16 items is recommended for the assessment of olfactory ability in clinical routine. For a more precise examination of olfaction (e.g., in rhinology centers or in studies), the threshold value and discrimination ability should also be determined, whereby the threshold value is the more important of the two parameters [72]. 

### Combination of mepolizumab with other biologics in chronic rhinosinusitis with nasal polyps 

Type 2 inflammatory comorbidities may respond very differently to one biologic, so in rare cases a combination of several type 2 biologics may be necessary. If, in close interdisciplinary cooperation, no single biologic can be identified that leads to control of comorbid type 2 diseases in a single patient, the comorbidities should be treated independently of each other in accordance with the guidelines. For example, patients occasionally show a very good response of CRSwNP to mepolizumab, while control of their asthma cannot be achieved with it and vice versa. 

In such a case, it may be necessary to provide the indication for a specific biologic for each disease separately according to the respective approvals so that ultimately a combination therapy consisting of several biologics is created [73]. In such a case, it is important that each specialist group provides the indication for both CRSwNP and asthma in accordance with the approval in-label. These are very individual treatment decisions. To date, there are no general recommendations for combining different biologics. There is also no reason for safety concerns [73]. However, such multiple treatments have not yet been recorded systematically enough to be able to make valid statements. Data from biologics registries will hopefully soon provide scientific findings. 

## Discussion 

With the European approval of mepolizumab as an add-on therapy with intranasal GCS for the treatment of adults with severe CRSwNP that cannot be adequately controlled with systemic GCS and/or surgery, an IL-5-addressing biologic has been available for the treatment of CRSwNP and can be prescribed and reimbursed in Germany since 2021 [74]. Mepolizumab represents an important advance in the treatment of CRSwNP and was urgently needed for patients with this disease, as it avoids the adverse effects of the previously required use of systemic GCS. With additional approvals for omalizumab and dupilumab, biologics may open up the possibility of implementing the principle of “personalized medicine” for CRSwNP [33]. 

Since biologics are cost-intensive drugs that in principle require a lifelong therapy, compliance with the economic efficiency requirements are of particular importance for their use in the German healthcare system. Doctors must provide the necessary, sufficient and appropriate services at the lowest possible cost to the health insurance funds [12, 75]. This will be the case if therapeutic alternatives have already been used unsuccessfully or are not available due to side effects or, particularly in case of surgery, unacceptable burdens or risks [12, 75]. Comparative studies on the cost-effectiveness of sinus surgery versus mepolizumab therapy for CRSwNP are now also available [76]. However, prospective cost-benefit analyses are still lacking. We have commented in detail on the use of the various biologics with approval in the indication area of CRSwNP and developed documentation forms that enable in-label use [12, 14, 15]. Several years of experience from the treatment of a large number of patients are now available, which make it necessary to provide corresponding recommendations for follow-up documentation during the course of treatment, which are summarized in this position paper. 

A response of CRSwNP to treatment with mepolizumab can usually be expected within 4 – 6 months. In this case – assuming good tolerability – treatment should be continued unchanged. In the event of only a partial response, treatment can also be continued, but reassessment is then required after 12 months of therapy at the latest. A combination of biologic treatment with short-term therapy with systemic GCSor surgery is possible, but the greatest possible restraint should be exercised, especially when using systemic GCS. The continuation of intranasal GCS application together with the biologic is always expressly recommended, even if the therapy is clearly successful, as mepolizumab was approved as an add-on therapy to intranasal GCS. If the therapeutic response remains insufficient after 12 months, treatment should be discontinued, but a switch to another biologic may be considered. If there is a very good response to treatment with appropriate control of CRSwNP, treatment can be paused after a few years with continued basic therapy with intranasal GCS [78]. However, as current knowledge suggests that disease control is then likely to be gradually lost, close monitoring of the patient is necessary and if a clinically relevant worsening of symptoms occurs, the previously successful treatment should be restarted. An extension of the treatment intervals appears medically possible, but does currently not correspond to the approved application intervals. 

Due to the primarily inflammatory pathophysiology of CRSwNP and the disproportionately higher costs of biologics compared to other treatment options, it can be assumed that intranasal GCS will continue to be the basic therapeutic agents in the future and that surgical treatments will remain indispensable. The authors would like to point out that the recommendations given here are consensus recommendations from our group of experts – there is not yet a sufficient scientific data basis for all the recommendations made, but this is expected in the coming years, especially from registry studies. We will therefore continuously adapt these recommendations to the current state of scientific knowledge. 

## Funding 

No funding. 

## Conflict of interest 

B. Wollenberg has received honoraria and/or research funding from MSD, Sanofi, AstraZeneca, Novartis, BMS Adboard outside the present work. 

J. Hagemann states that he has received payments for lectures and fees for advisory boards from the companies Sanofi Aventis, Novartis Pharma GmbH, and GlaxoSmithKline. 

A.M. Chaker provides consulting services (e.g., Advisory Boards, DSMBs), lectures, or other activities via the Technical University of Munich (TUM) or has conducted clinical studies or received research funding via TUM from: Allergopharma, ALK-Abello, AstraZenecaa, Bencard/Allergen Therapeutics, GSK, HAL Allergy, Immunotek, Novartis, SanofiGenzyme and Regeneron, Zeller AG, EIT Health, BMBF. AMC is also an officer of EUFOREA, EAACI, AeDA, and DGAKI. 

H. Olze has received honoraria and/or research funding from F. Hoffmann-La Roche Ltd., Sanofi-Aventis Deutschland GmbH, AstraZeneca GmbH, GlaxoSmithKline GmbH & Co KG, and Novartis Pharma GmbH. 

L. Klimek reports grants and/or honoraria from Allergopharma, MEDA/Mylan, HAL Allergie, ALK Abelló, LETI Pharma, Stallergenes, Quintiles, Sanofi, ASIT Biotech, Lofarma, Allergy Therapeut., AstraZeneca, GSK, Inmunotk, outside the submitted work; and membership in the following organizations: AeDA, DGHNO, German Academy for Allergology and Clinical Immunology, HNO-BV GPA, EAACI. 

U. Förster-Ruhrmann received honoraria for lectures from Novartis, AstraZeneca, Sanofi. and GSK outside the present work. 

S. Strieth reports grants from the German Research Foundation (DFG), Bonn, grants from the Head and Neck Tumor Research Foundation, Wiesbaden, grants and non-financial support from MED-EL AG, Innsbruck, personal fees from Auris Medical, Basel, personal fees from Merck Serono, Darmstadt, personal fees from Otonomy, Inc, San Diego (USA), personal fee Nordmark Arzneimittel, Uetersen, grant Andreas Fahl Medizintechnik-Vertrieb, Cologne, grant Atos Medical, Troisdorf, grant Tracoe Medical, Nieder-Olm, grants from Heimomed Heinze, Kerpen, grants from Bromepithetik, Heidelberg, grants from Fresenius Kabi, Bad Hersfeld, personnel fee from Sonofi Genzyme, Berlin, personal fee from ALK-Abelló Arzneimittel, Hamburg, outside the submitted work. 

M. Cuevas has received honoraria and/or non-financial support from Novartis, Sanovi-Aventis, Allergopharma, HAL Allergy, Leti Pharma, AstraZeneca, GlaxoSmithKline, ALK Abelló, Bencard Allergy, Stallergenes, and Roxall outside the submitted work and reports memberships with the following organizations: AeDA, DGHNO. 

A.G. Beule has received honoraria for lectures, consulting, or research activities from Allakos, AstraZenecaa, BMS, GSK, Medtronic, MSD, Novartis, Olympus, Pharmalog, Pohl Boskamp, and Sanofi Aventis outside the present work. 

O. Guntinas-Lichius has received honoraria from MED-EL, Merck, Novartis, MEDICE, and Merz outside the present work; and he is a member of the following organizations: DGHNO, BVHNO. 

T.K. Hoffmann participates in honorary advisory boards of the companies Merck, MSD, and BMS, but outside the scope of this work. 

C. Bachert received honoraria/research funding from the companies Sanofi, GSK, Novartis, Astra Zeneca, and ALK outside the present work. 

H. Wrede reports lecture fees from Allergopharma, MEDA/Mylan, HAL Allergie, LETI Pharma, Stallergenes, Sanofi, Lofarma, Allergy Therapeut., GSK outside the submitted work; and membership of the following organizations: AeDA, HNO-BV. 

T. Stöver has received research and study funding as well as fees for lectures and/or consultancy work from MED-EL Elektromedizinische Geräte Deutschland GmbH and Cochlear Deutschland GmbH & Co. KG outside the submitted work. He is a member of the DGHNO-KHC, the expert committee on medical devices and in-vitro diagnostics on behalf of the EU, the advisory board of the Hörzentrum Oldenburg GmbH, the task force ‘Living Practice Guidelines’, the advisory board of the Lower Saxony Center for Biomathematics, as chairman of the advisory board of the Friedberg Foundation for Hearing and Speech Promotion and as co-editor of the journal Laryngo-Rhino-Otology. 

C. Beutner on fees from GSK, Sanofi and Novartis, ALK Abello outside the present work. 

M. Laudien supported and received support, lecture, and consulting fees in the last 5 years from: Olympus Deutschland GmbH, Olympus Europa SE & CO. KG, Novartis Pharma GmbH, Sanofi-Aventis Deutschland GmbH, Brainlab Sales GmbH, GlaxoSmithKline GmbH & Co KG and the John Grube Foundation outside the present work. 

M. Gröger reports grants and lecture fees from Sanofi, Novartis, AstraZeneca, and GSK outside the submitted work. 

C. Bergmann reports on grants and fees from GlaxoSmithKline (GSK), Sanofi Aventis, Bencard Allergy GmbH/Allergy Therapeutics, HAL Allergie GmbH/HAL Allergy Holding BV outside the present work. 

O. Pfaar receives honoraria and/or study funding from ALK-Abelló, Altamira, Allergopharma, Stallergenes Greer, HAL Allergy Holding B.V./HAL Allergie, AAAAI, Bencard Allergy/Allergy Therapeutics, Lofarma, Biomay, Circassia, ASIT Biotech Tools S.A., Danish Consultation, Laboratorios LETI/LETI Pharma, MEDA Pharma/MYLAN, Anergis S.A., Mobile Chamber Experts, Indoor Biotechnologies, GlaxoSmithKline, Astellas Pharma Global, EUFOREA, ROXALL, Novartis, Sanofi-Aventis and Sanofi-Genzyme, Med Update Europe, streamedup!, Pohl-Boskamp, Inmunotek S.L., Wiley and Sons, Paul Martini Foundation, Regeneron Pharmaceuticals Inc, RG Aerztefortbildung, Firma Meinhardt, PneumoLIVE, Institut für Disease Management, Deutsche Forschungsgesellschaft, Springer, Thieme, AstraZeneca, Deutsche Allergie-Liga, AeDA, IQVIA Commercial, Ingress Health, Wort&Bild Verlag, Verlag ME, Procter&Gamble, Alfried-Krupp Krankenhaus, all outside the present work; and he is a member of the board/excom of the EAACI and a member of the extended board of the DGAKI. He is also the main author or co-author of various guidelines and position papers in allergology and rhinology. 

A.S. Hoffmann has received honoraria from GSK, Sanofi, and Novartis for lectures and advisory boards outside this work. 

T. Hildenbrand reports on lecture fees from AstraZeneca and Novartis outside the present work. 

R. Weber has received fees for lecturing and consulting activities from GSK, Infectopharm, KARL STORZ SE & Co KG, NMP, Sanofi, Sidroga-Pharma, and Stryker. 

W. Schlenter, S. Becker, F. Bärhold, T. Deitmer, H.J. Welkoborsky, S. Dazert, T. Huppertz, C.A. Hintschich, J. Zuberbier, C. Rudack, J. Gosepath, P. Werminghaus, F. Klimek and I. Casper have no conflicts of interest in connection with the present work. 

**Table 1.** Objectifiable parameters for a response to mepolizumab therapy after 6 months (at least 1 parameter should be fulfilled) (modified from [6]).

Nasal obstruction: improvement of the nasal congestion score (0 – 3) by > 0.5 or improvement of objective tests (e.g., increase in nasal inspiratory peak flow or nasal volume flow in active anterior rhinomanometry by > 20 L/min, reduction in resistance)
Nasal polyp score (NPS): Reduction of the endoscopically determined NPS (0 – 8) by > 1 score point compared to the initial value
SNOT-22/quality of life: reduction in SNOT-22 score (0 – 110) by > 8.9 (validated minimum clinically relevant difference)
Symptomatology in visual analog scale (VAS): reduction in total VAS symptoms (0 – 10 score points) by > 2
Smelling ability: improvement in the 16-point Sniffin‘ Sticks identification test by > 3 points (validated minimum clinically relevant difference)

**Table 2.** Objectifiable parameters for an evaluation of long-term mepolizumab therapy (> 12 months) (modified from [6]).

All symptoms are only moderately pronounced or at least improved compared to the status before the start of therapy
Total nasal polyp score < 4 (added on both sides)
Nasal congestion score < 2 (the nasal passage allows almost normal breathing at rest)
Visual analog scale total symptoms < 5
SNOT-22 value < 30
Chronic rhinosinusitis with nasal polys (CRSwNP) should not currently require the administration of systemic glucocorticosteroids or surgery for CRSwNP (except surgery to remove mechanical obstructions such as synechiae, mucoceles, etc.).
